# Dysregulated alveolar epithelial cell progenitor function and identity in Hermansky-Pudlak syndrome

**DOI:** 10.1172/jci.insight.183483

**Published:** 2024-12-19

**Authors:** Joanna Y. Wang, Sylvia N. Michki, Sneha Sitaraman, Brandon J. Banaschewski, Reshma Jamal, Jason J. Gokey, Susan M. Lin, Jeremy B. Katzen, Maria C. Basil, Edward Cantu, Jonathan A. Kropski, Jarod A. Zepp, David B. Frank, Lisa R. Young

**Affiliations:** 1 Division of Pulmonary, Allergy, Critical Care, and Sleep Medicine, Department of Medicine, University of Pittsburgh School of Medicine, Pittsburgh, Pennsylvania, USA.; 2 Division of Pulmonary, Allergy, and Critical Care, Department of Medicine, Perelman School of Medicine, University of Pennsylvania, Philadelphia, Pennsylvania, USA.; 3 Division of Cardiology, Department of Pediatrics, and; 4 Division of Pulmonary and Sleep Medicine, Department of Pediatrics, Children’s Hospital of Philadelphia, Philadelphia, Pennsylvania, USA.; 5 Division of Allergy, Pulmonary, and Critical Care Medicine, Department of Medicine, Vanderbilt University, Nashville, Tennessee, USA.; 6 Lung Biology Institute and; 7 Penn Cardiovascular Institute, University of Pennsylvania, Philadelphia, Pennsylvania, USA.; 8 Department of Surgery, Perelman School of Medicine, University of Pennsylvania, Philadelphia, Pennsylvania, USA.

**Keywords:** Cell biology, Pulmonology, Fibrosis, Genetic diseases, Lysosomes

## Abstract

Hermansky-Pudlak syndrome (HPS) is a genetic disorder of endosomal protein trafficking associated with pulmonary fibrosis in specific subtypes, including HPS-1 and HPS-2. Single-mutant HPS1 and HPS2 mice display increased fibrotic sensitivity while double-mutant HPS1/2 mice exhibit spontaneous fibrosis with aging, which has been attributed to HPS mutations in alveolar epithelial type II (AT2) cells. We utilized HPS mouse models and human lung tissue to investigate mechanisms of AT2 cell dysfunction driving fibrotic remodeling in HPS. Starting at 8 weeks of age, HPS mice exhibited progressive loss of AT2 cell numbers. HPS AT2 cell function was impaired ex vivo and in vivo. Incorporating AT2 cell lineage tracing in HPS mice, we observed aberrant differentiation with increased AT2-derived alveolar epithelial type I cells. Transcriptomic analysis of HPS AT2 cells revealed elevated expression of genes associated with aberrant differentiation and p53 activation. Lineage-tracing and organoid-modeling studies demonstrated that HPS AT2 cells were primed to persist in a Keratin-8–positive reprogrammed transitional state, mediated by p53 activity. Intrinsic AT2 progenitor cell dysfunction and p53 pathway dysregulation are mechanisms of disease in HPS-related pulmonary fibrosis, with the potential for early targeted intervention before the onset of fibrotic lung disease.

## Abstract 2

Alveolar epithelial cells exhibit dysregulated progenitor cell function with impaired proliferation and aberrant differentiation in the setting of protein trafficking defects in Hermansky-Pudlak syndrome pulmonary fibrosis.

## Introduction

Hermansky-Pudlak syndrome (HPS) is an autosomal recessive disorder of endosomal protein trafficking. At birth, patients with HPS do not exhibit pulmonary disease, but progressive pulmonary fibrosis typically develops in young adulthood in HPS-1 and HPS-4 and in childhood in HPS-2 (1). Notably, pulmonary fibrosis has not been reported in HPS-3, which is the second most common HPS subtype after HPS-1. We originally demonstrated that HPS mutations specifically in the alveolar epithelium underlie fibrotic susceptibility (2). Evidence suggests that dysfunctional alveolar epithelial type II (AT2) cells, which act as stem cells within the alveolus and produce and store surfactant, drive fibrotic remodeling in other genetic and sporadic forms of pulmonary fibrosis (3–5). In HPS-related pulmonary fibrosis (HPS-PF), studies from several groups have implicated apoptosis, impaired autophagy, and mitochondrial dysfunction in AT2 cells as potential contributing pathways (2, 6–9). Despite these advances, the development of effective, targeted therapies for HPS-PF has been limited by an incomplete understanding of the consequences of lysosomal and endosomal trafficking defects on AT2 cell function, as well as targetable mechanisms underlying HPS AT2 cell dysfunction. In addition, it remains unclear whether there are common final pathways of AT2 cell dysfunction shared across sporadic and genetic fibrotic lung diseases to inform the generalizability of therapies for pulmonary fibrosis that are currently available or in development.

In this study, we examined HPS AT2 cells over time to characterize the cellular and molecular mechanisms that drive AT2 cell dysfunction and their contribution to the fibrotic response. We utilized 5 murine models of HPS, including the double-mutant HPS1/2 murine model, which exhibits spontaneous progressive fibrosis with aging, as well as the single-mutant HPS1 and HPS2 models, which best reflect human disease genetics while exhibiting fibrotic sensitivity without spontaneous fibrosis (Table 1) (8, 10–12). We report that in HPS genotypes associated with pulmonary fibrosis, impaired AT2 cell progenitor function occurred early, brought about in part by p53 activation. Starting at 8 weeks of age in HPS mice, we show dysregulated maintenance of the alveolar epithelium, with progressive loss of AT2 cells in association with impaired proliferation, aberrant proliferation, and persistence of a Keratin-8–positive (Krt8^+^) reprogrammed transitional cell state. We also demonstrate the presence of senescent and KRT8^+^ epithelial cells in lung tissue from a patient with HPS-1. Our findings reveal a potentially novel therapeutic target in HPS-PF with the potential for early intervention and suggest the existence of convergent mechanisms of disease between HPS-PF and other sporadic and genetic fibrotic lung diseases.

## Results

### Loss of AT2 cells in HPS mice starting at 8 weeks of age.

**Figure 1:**
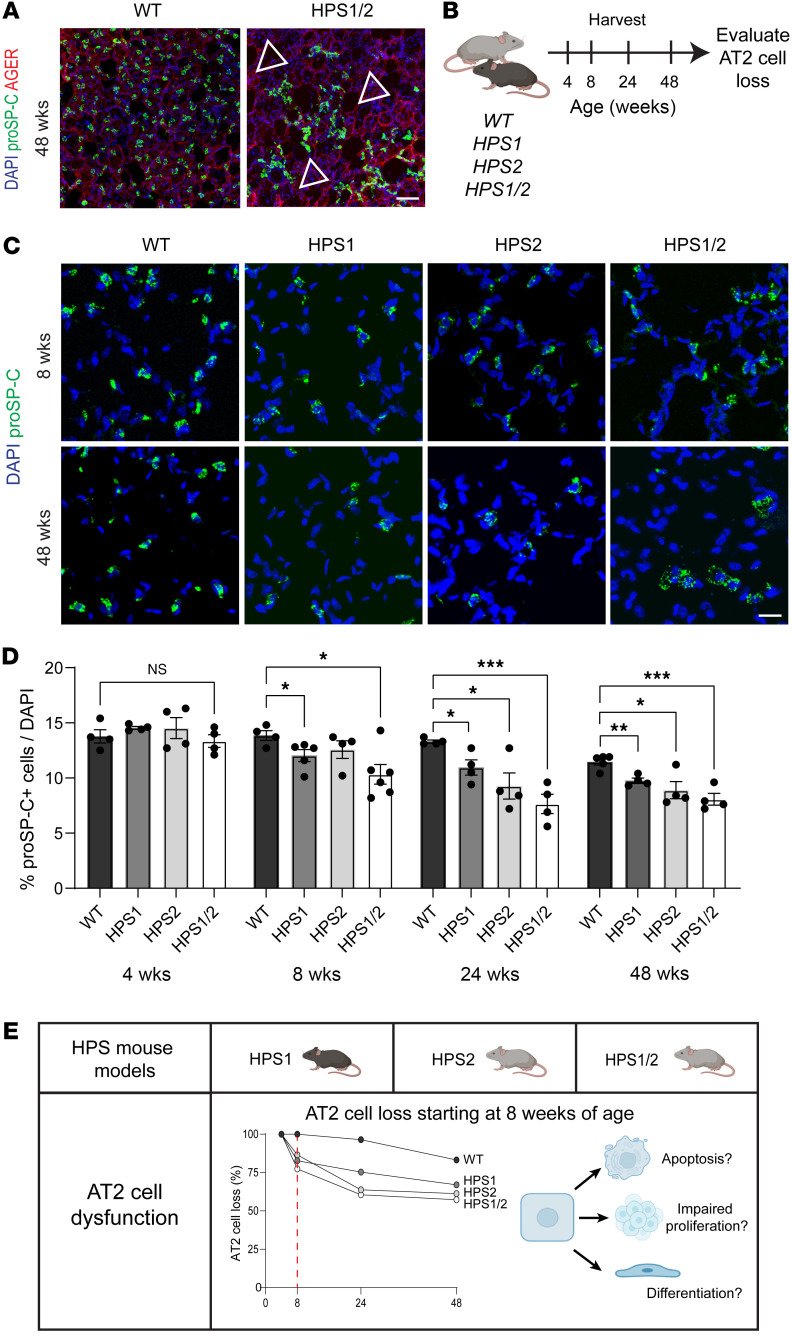
Loss of AT2 cells in HPS mice starting at 8 weeks of age. (**A**) IF staining of whole-mount lung issue for pro-SPC and AGER in WT and HPS1/2 mice at 48 weeks of age. Arrows indicate regions of AT2 cell loss. (**B**) Schematic to evaluate AT2 cell loss in WT, HPS1, HPS2, and HPS1/2 mice over time. (**C**) Staining of paraffin-embedded lung tissue for proSP-C in WT, HPS1, HPS2, and HPS1/2 mice at 8 and 48 weeks of age. (**D**) Quantification of percentage of proSP-C^+^ cells as a percentage of total cells (by DAPI) in WT, HPS1, HPS2, and HPS1/2 mice at 4, 8, 24, and 48 weeks of age. (**E**) Schematic of AT2 cell loss in HPS mice and possible etiologies. DAPI stains nuclei (blue). All quantification data are represented as mean ± SEM. Statistics using 2-tailed unpaired Student’s *t* tests: adjusted * *P* < 0.05; ** *P* < 0.01, *** *P* < 0.001 after Benjamini-Hochberg correction for multiple comparisons. *n* = 4–6 per group per time point. Scale bars in **A**, 50 μm; **C**, 20 μm. Schematics created with BioRender.com.

We first verified that double-mutant HPS1/2 mice developed spontaneous, progressive fibrosis with aging as previously described (Supplemental Figure 1, A–D; supplemental material available online with this article; https://doi.org/10.1172/jci.insight.183483DS1) (11–13). We then utilized immunofluorescence (IF) for the AT2 cell marker, prosurfactant protein C (proSP-C), and the alveolar epithelial type I (AT1) cell marker, AGER, to examine the alveolar epithelium in aged HPS1/2 mice compared with wild-type (WT) mice at 48 weeks of age. In addition to clusters of AT2 cell hyperplasia, we observed regions of near-total loss of proSP-C^+^ AT2 cells in HPS1/2 mice (Figure 1A). To evaluate the time course of AT2 cell loss, we utilized IF for proSP-C to quantify AT2 cells in WT and HPS1/2 mice at 4, 8, 24, and 48 weeks of age (Figure 1, B–D). Starting at 8 weeks of age, we found a significant decrease in the percentage of proSP-C^+^ AT2 cells per total DAPI-positive cells in HPS1/2 mice, with 10.3% ± 0.4% in HPS1/2 mice compared with 13.9% ± 0.3% in WT (adjusted *P* = 0.02) (Figure 1D). These percentages corresponded to a mean 31.0 ± 10.2 cells per high-powered field (hpf) in HPS1/2 mice and 56.8 ± 10.4 AT2 cells per hpf in WT mice (*P* < 0.001). There was progressive loss of AT2 cells with aging in HPS1/2 mice, which exhibited 8.1% ± 0.5% proSP-C^+^ cells at 48 weeks of age (adjusted *P* = 0.001), as well as WT mice with aging, with 11.5% ± 0.2% at 48 weeks of age (adjusted *P* = 0.002) (Figure 1D).

As HPS1/2 mice contain mutations in both *Hps1* and *Ap3b1* (*Hps2*), we next assessed HPS1 and HPS2 mice to evaluate if the phenotype of AT2 cell loss was dependent on the combinatorial experimental HPS model or also occurred in mice with disruption in a single HPS gene. Significant AT2 cell loss was also seen in single-mutant HPS1 (adjusted *P* < 0.0001) and HPS2 mice (adjusted *P* = 0.037; Figure 1, C and D). The difference in AT2 cell numbers was detected starting at 8 weeks of age in HPS1 mice and at the 24-week time point for HPS2 mice. To account for the possibility of immune cells and other cell types present in HPS mice that could alter the total cell count and contribute to lower percentages of proSP-C^+^ cells, we quantified the percentage of proSP-C^+^ cells per total epithelial cells as denoted by NKX2.1 staining (Supplemental Figure 2, A–D). There was a significant decrease in the percentage of proSP-C^+^NKX2.1^+^ cells per total epithelial cells by NKX2.1 staining in HPS1 compared with WT mice at 8 weeks of age (72.2% ± 3.2% vs. 78.2% ± 3.5%, *P* = 0.046) and in HPS2 compared with WT mice at 24 weeks of age (65.0% ± 2.4% vs. 76.1% ± 3.9%, *P* = 0.003) (Supplemental Figure 2, A–D). In contrast, in HPS3 mice, a subtype that has not been associated with pulmonary fibrosis, AT2 cell numbers were comparable to WT at 8 and 24 weeks of age (Supplemental Figure 3, A and B). Furthermore, in transgenic epithelially corrected HPS2 (HPS2 TG) mice, AT2 cell numbers were significantly higher compared with HPS2 mice at 24 weeks of age (Supplemental Figure 3, A and C).

Together, these data show that starting at 8 weeks of age, prior to the onset of fibrosis, there is progressive loss of AT2 cells in HPS1, HPS2, and HPS1/2 mice, the HPS subtypes associated with fibrotic susceptibility (Figure 1E). We hypothesized that this loss could result from increased apoptosis, impaired proliferation, or aberrant differentiation (Figure 1E). Notably, no TUNEL-positive apoptotic AT2 cells were detected in WT or HPS mice across the same time points (Supplemental Figure 4A), suggesting that AT2 cell apoptosis was a rare event in HPS mice, apoptotic cells were rapidly cleared, or loss of AT2 cells was occurring predominantly through other mechanisms.

### Impaired AT2 cell response after acute influenza injury in HPS mice.

**Figure 2:**
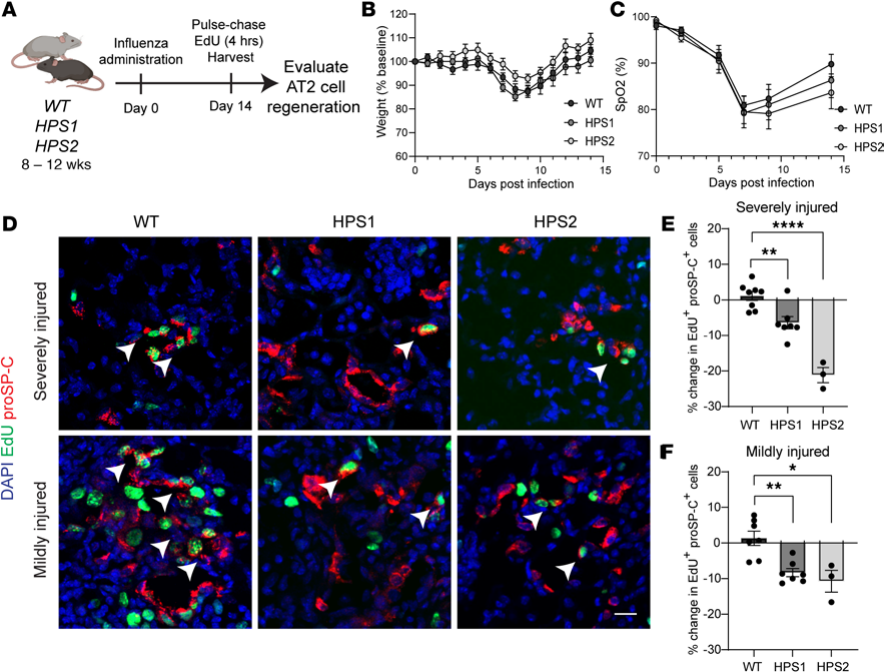
Impaired regeneration of HPS AT2 cells after acute influenza injury. (**A**) Schematic of influenza experiment. (**B** and **C**) Mean change in (**B**) weight and (**C**) oxygen saturation (SpO_2_) in WT, HPS1, and HPS2 mice from 0 to 14 dpi. (**D**) IF staining of lung tissue for proSP-C and EdU in WT, HPS1, and HPS2 mice at 14 dpi in severely and mildly injured regions. Arrows indicate proliferating AT2 cells (EdU^+^proSP-C^+^). (**E** and **F**) Quantification of the percentage difference in proliferating AT2 cells (EdU^+^proSP-C^+^) in (**E**) severely injured and (**F**) mildly injured regions at 14 dpi in HPS1 and HPS2 mice relative to WT. DAPI stains nuclei (blue). All quantification data are represented as mean ± SEM. Statistics using 2-tailed unpaired Student’s *t* tests: adjusted * *P* < 0.05, ** *P* < 0.01, **** *P* < 0.0001 after Benjamini-Hochberg correction for multiple comparisons. *n* = 3–10 per group. All scale bars, 20 μm. Schematic created with BioRender.com.

To evaluate whether impaired proliferation contributes to AT2 cell loss, we employed an influenza model to evaluate AT2 cell function in the context of regeneration after physiological injury (Figure 2A) (14, 15). Because AT2 cell loss occurred in HPS1, HPS2, and HPS1/2 mice, we utilized HPS1 and HPS2 mice at 8 weeks of age for the subsequent proliferation studies, as these mice best reflect human disease genetics. Weight change and oxygen saturation decreased similarly in WT, HPS1, and HPS2 mice during the first 10 days postinfection (dpi) with H1N1 PR8 influenza; subsequently, oxygen saturations trended lower in HPS1 and HPS2 mice, though no mortality was observed in any group (Figure 2, B and C). To ensure appropriate quantification and comparison across groups with the heterogeneous nature of influenza-induced lung injury, a computational image analysis approach was used to cluster image pixels based on H&E staining to define 3 regions: severely injured, mildly injured, and unaffected (Supplemental Figure 5A). There was no significant difference in the percentages of these regions or in the area formed by KRT5-expressing cells across all groups at 14 dpi (Supplemental Figure 5, A–E).

To examine epithelial regeneration after injury, we examined proliferating AT2 (5-ethynyl-2′-deoxyuridine–positive [EdU^+^], proSP-C^+^) cells in HPS1 and HPS2 as compared with WT mice after acute influenza infection (Figure 2, D–F). The absolute numbers of EdU^+^proSP-C^+^ cells were greatest in severely and mildly injured regions in WT mice; there were fewer EdU^+^proSP-C^+^ cells in the corresponding regions in HPS1 and HPS2 mice (mean ± SEM 97.5 ± 8.9 EdU^+^proSP-C^+^ cells in WT mice, 69.7 ± 12.6 in HPS1 mice, and 22.0 ± 3.2 in HPS2 mice for severely injured regions; 72.0 ± 2.5 EdU^+^proSP-C^+^ cells in WT mice, 55.3 ± 9.5 in HPS1 mice, and 32.5 ± 0.5 in HPS2 mice in mildly injured regions). In unaffected areas where histologic evidence of injury was not observed, EdU^+^proSP-C^+^ cells were extremely rare (not shown). Given the heterogeneous nature of influenza injury and previously observed AT2 cell loss in naive HPS mice, we quantified the difference in the percentages of EdU^+^proSP-C^+^ cells in WT, HPS1, and HPS2 mice. A decrease in the percentage of proliferating AT2 cells was observed in both severely injured and mildly injured regions in both HPS models at 14 dpi compared with WT (Figure 2, D–F). In severely injured regions, there was a 6.4% ± 4.5% decrease in EdU^+^proSP-C^+^ cells in HPS1 mice (adjusted *P* = 0.0033) and a 21.2% ± 3.7% decrease in HPS2 mice (adjusted *P* < 0.0001) compared with WT (Figure 2, D and E). In mildly injured regions, there was an 8.3% ± 3.0% reduction in EdU^+^proSP-C^+^ cells in HPS1 mice (adjusted *P* = 0.0012) and a 10.8% ± 5.3% decrease in HPS2 mice (adjusted *P* = 0.011) compared with WT (Figure 2, D and F). Overall, these data demonstrate impaired HPS AT2 cell proliferation in the setting of acute influenza injury.

### Intrinsic HPS AT2 cell proliferation defect ex vivo and in vivo.

**Figure 3:**
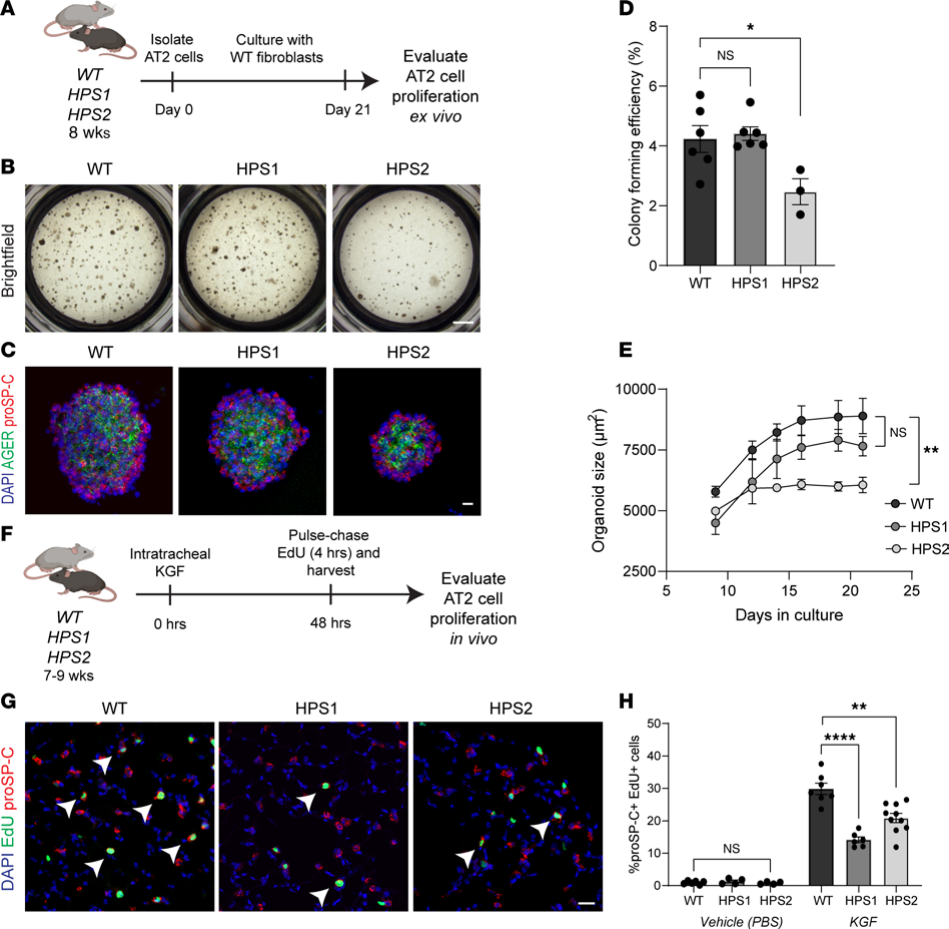
Impaired proliferation of HPS AT2 cells ex vivo and in vivo. (**A**) Schematic of lung organoid culture system. (**B**) Bright-field imaging and (**C**) IF staining of lung organoids for AGER and proSP-C generated with AT2 cells isolated from WT, HPS1, or HPS2 mice with WT fibroblasts at day 21 of culture. (**D**) Quantification of WT, HPS1, and HPS2 organoid CFE at day 21 of culture. (**E**) Quantification of WT, HPS1, and HPS2 organoid size from day 9 to day 21 of culture. (**F**) Schematic of keratinocyte growth factor (KGF) experiment. (**G**) IF staining of lung tissue for proSP-C and EdU in WT, HPS1, and HPS2 mice 48 hours after KGF administration. Arrows indicate proliferating AT2 cells (EdU^+^proSP-C^+^). (**H**) Quantification of the percentage of proliferating AT2 cells (EdU^+^proSP-C^+^) in WT, HPS1, and HPS2 mice treated with vehicle (PBS) versus KGF. DAPI stains nuclei (blue). All quantification data are represented as mean ± SEM. Statistics using 2-tailed unpaired Student’s *t* tests: adjusted * *P* < 0.05, ** *P* < 0.01, **** *P* < 0.0001 after Benjamini-Hochberg correction for multiple comparisons. For **D** and **E**, each point represents the average of at least 3 replicate wells from 1 mouse for a total of *n* = 3–6 mice per group. For **H**, *n* = 4–10 per group per treatment. Scale bars in **B** 1 mm; in **C** and **G** 20 μm. Schematics created with BioRender.com.

To evaluate whether this impaired HPS AT2 cell proliferation was due to cell-intrinsic defects in progenitor function and proliferative capacity, we first employed an ex vivo organoid model (15). We generated lung organoids with WT primary outgrowth lung fibroblasts and AT2 cells from WT, HPS1, and HPS2 mice at 8 weeks of age (Figure 3A). Compared with lung organoids generated with WT AT2 cells, those with HPS2 AT2 cells were significantly smaller in size (adjusted *P* = 0.002) and displayed significantly decreased colony-forming efficiency (CFE), 2.5% ± 0.4% compared with 4.2% ± 0.4% in WT organoids (adjusted *P* = 0.043), over 21 days of culture (Figure 3, B–E). In contrast, organoids generated from HPS1 AT2 cells demonstrated comparable size and growth compared to those generated with WT AT2 cells (Figure 3, B–E).

To evaluate intrinsic AT2 cell proliferative capacity in vivo, we administered an epithelial cell–specific mitogen, keratinocyte growth factor (KGF), to WT, HPS1, and HPS2 mice at 8 weeks of age and quantified the percentage of EdU^+^proSP-C^+^ cells 48 hours after KGF administration (Figure 3F). The percentage of proliferating AT2 cells was significantly reduced in HPS1 (14.2% EdU^+^proSP-C^+^ cells, adjusted *P* < 0.0001) and HPS2 mice (20.9% ± 4.5% EdU^+^proSP-C^+^ cells, adjusted *P* = 0.001) compared with WT (29.9% ± 4.6% EdU^+^proSP-C^+^ cells) (Figure 3, G and H). Low rates of AT2 cell proliferation were observed in WT, HPS1, and HPS2 mice administered vehicle (Figure 3H). Overall, these data demonstrate an intrinsic proliferative defect in HPS AT2 cells ex vivo and in vivo.

### Lineage tracing of HPS AT2 cells reveals spontaneous aberrant differentiation at 8 weeks of age.

**Figure 4:**
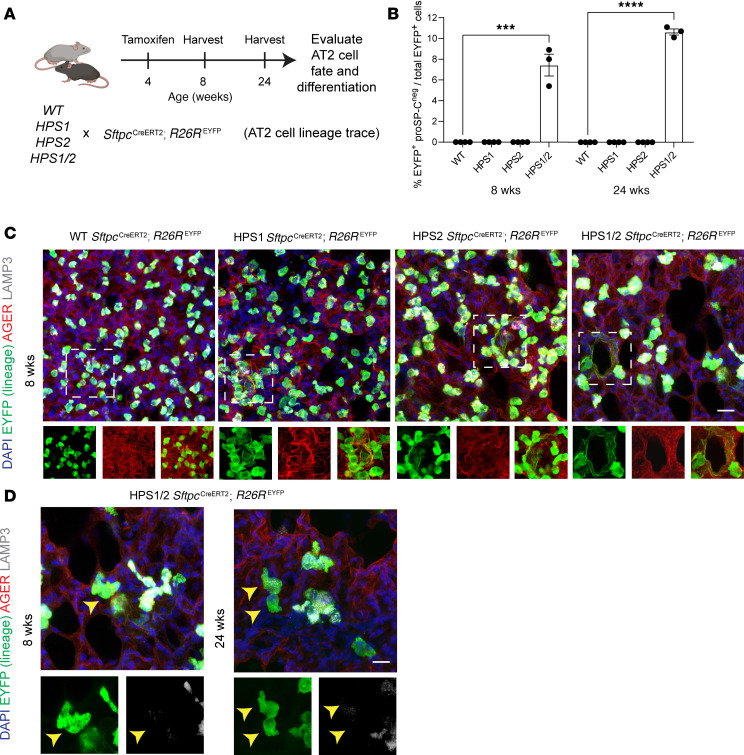
Lineage tracing of AT2 cells in HPS mice demonstrates aberrant differentiation at 8 weeks of age. (**A**) Schematic of lineage labeling AT2 cells in WT, HPS1, HPS2, and HPS1/2 *Sftpc*^CreERT2/+^ *R26R*^EYFP/+^ mice. (**B**) From images in Supplemental Figure 6A, quantification of EYFP^+^proSP-C^–^ cells as a percentage of EYFP^+^ lineage cells in WT, HPS1, HPS2, and HPS1/2 *Sftpc*^CreERT2/+^ *R26R*^EYFP/+^ mice at 8 and 24 weeks of age. (**C**) IF staining of whole-mount lung tissue for EYFP, AGER, and LAMP3 in WT, HPS1, HPS2, and HPS1/2 *Sftpc*^CreERT2/+^ *R26R*^EYFP/+^ mice at 8 weeks of age. Dashed line boxes represent squamous EYFP^+^AGER^+^LAMP3^–^ AT2-derived AT1 cells with AGER and EYFP and merged images shown below. (**D**) IF staining of HPS1/2 *Sftpc*^CreERT2/+^ *R26R*^EYFP/+^ mice at 8 and 24 weeks of age, with arrows indicating cuboidal EYFP^+^AGER^–^LAMP3^–^ cells with EYFP and LAMP3 images shown below. HPS1/2 *Sftpc*^CreERT2/+^ *R26R*^EYFP/+^ 8-week images (**C**) and demonstrated again (**D**) capture simultaneous presence of squamous EYFP^+^AGER^+^LAMP3^–^ and cuboidal EYFP^+^AGER^–^LAMP3^–^ cells. DAPI stains nuclei (blue). All quantification data are represented as mean ± SEM. Statistics using 2-tailed unpaired Student’s *t* tests: *** *P* < 0.001; **** *P* < 0.0001. *n* = 3–4 per group per time point. Scale bars in **C** and **D**, 20 μm (inset boxes equal scale to main image). Schematic created with BioRender.com.

To verify the finding of loss of AT2 cells identified based on proSP-C expression and further investigate the etiology of AT2 cell loss by evaluating cell fate and differentiation, we utilized the *Sftpc*^CreERT2/+^ *R26R*^EYFP/+^ reporter to generate 4 additional lines of mice (WT, HPS1, HPS2, and HPS1/2 mice, each with *Sftpc*^CreERT2/+^ *R26R*^EYFP/+^) to perform lineage tracing of AT2 cells. Recombination with tamoxifen was induced at 4 weeks of age, prior to the time point when we observed onset of significant AT2 cell loss based on proSP-C immunostaining (Figure 4A). Quantification of the number of lineage-labeled AT2 cells (EYFP^+^) in paraffin-embedded sections verified significant loss of AT2 cells starting at 8 weeks of age in HPS1, HPS2, and HPS1/2 mice, with quantitative results similar to those obtained using proSP-C immunostaining (Supplemental Figure 6, A and B). HPS1/2 *Sftpc*^CreERT2/+^ *R26R*^EYFP/+^ mice exhibited 9.0% ± 0.8% lineage-labeled AT2 cells compared with 14.4% ± 0.8% in WT *Sftpc*^CreERT2/+^ *R26R*^EYFP/+^ mice (adjusted *P* < 0.001) at 8 weeks of age (Supplemental Figure 6, A and B). Loss of lineage-labeled AT2 cells was also seen at 8 weeks of age in HPS1 *Sftpc*^CreERT2/+^ *R26R*^EYFP/+^ mice (11.8% ± 0.2% EYFP^+^ cells, adjusted *P* = 0.003) and HPS2 *Sftpc*^CreERT2/+^ *R26R*^EYFP/+^ (12.9 ± 1.1% EYFP^+^ cells, adjusted *P* = 0.034) compared with *Sftpc*^CreERT2/+^ *R26R*^EYFP/+^ mice (Supplemental Figure 6, A and B).

In HPS1/2 *Sftpc*^CreERT2/+^ *R26R*^EYFP/+^ mice, we observed the emergence of lineage-labeled cells that did not express proSP-C (EYFP^+^proSP-C^–^), constituting 7.4% ± 1.8% of all lineage-labeled cells at 8 weeks of age and 10.6% ± 0.5% at 24 weeks of age (Figure 4B and Supplemental Figure 6A). In contrast, EYFP^+^proSP-C^–^ cells were not identified in WT, HPS1, or HPS2 *Sftpc*^CreERT2/+^ *R26R*^EYFP/+^ mice at 8 or 24 weeks of age (Figure 4B and Supplemental Figure 6A). We next proceeded to investigate the identity of these lineage-labeled cells and AT2-to-AT1 cell differentiation as a potential etiology of AT2 cell loss. To account for the squamous morphology of AT1 cells, we performed immunostaining of thicker 150 μm whole-mount lung sections. Utilizing this technique, lineage-labeled EYFP^+^AGER^+^LAMP3^–^ cells with the squamous morphology of AT1 cells were identified in the lungs of HPS1, HPS2, and HPS1/2 *Sftpc*^CreERT2/+^ *R26R*^EYFP/+^ mice, starting at 8 weeks of age (Figure 4C). No lineage-labeled AT1 cells were detected in WT *Sftpc*^CreERT2/+^ *R26R*^EYFP/+^ mice (Figure 4C). Notably, cuboidal lineage-labeled EYFP^+^AGER^–^LAMP3^–^ cells were also observed, but only in HPS1/2 *Sftpc*^CreERT2/+^ *R26R*^EYFP/+^ mice, starting at 8 weeks of age and persisting through 24 weeks of age (Figure 4D). These lineage-tracing results verify AT2 cell loss and reveal spontaneous and aberrant differentiation of AT2 cells in HPS mice starting at 8 weeks of age.

### Transcriptomic signatures of senescence and aberrant differentiation are enriched in HPS AT2 cells.

**Figure 5:**
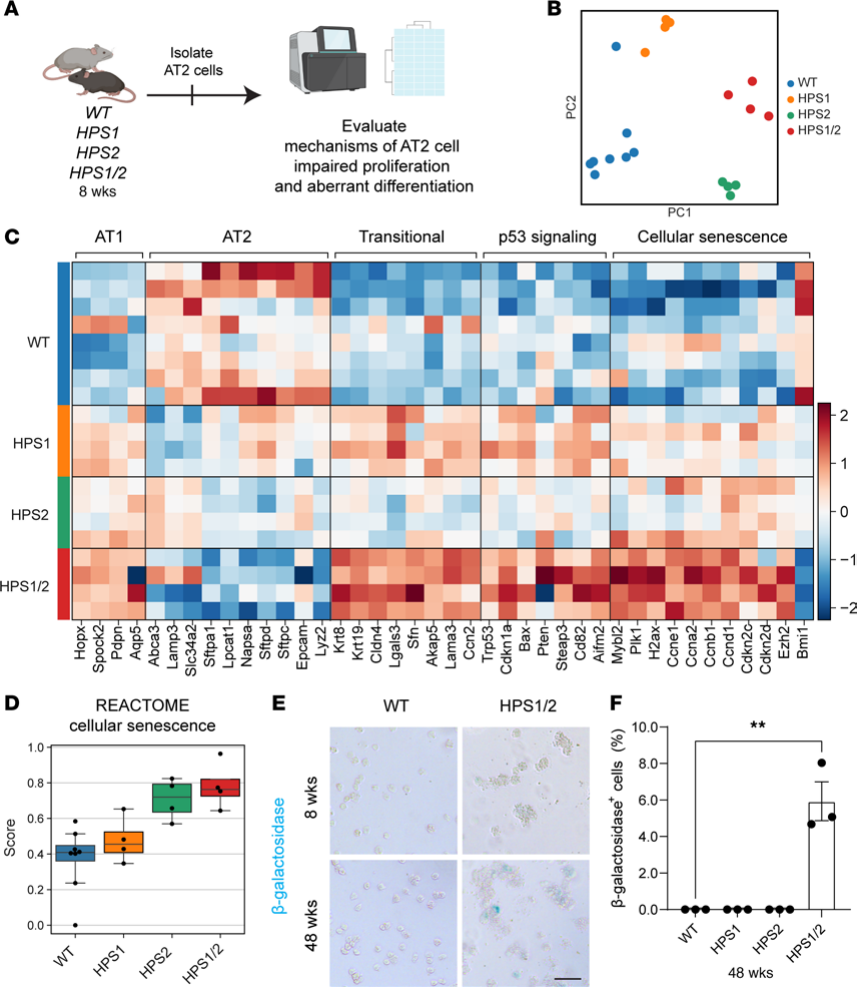
Transcriptomic analysis suggests aberrant differentiation and p53-mediated senescence in HPS AT2 cells. (**A**) Experimental schematic for bulk mRNA sequencing of HPS AT2 cells. (**B**) Principal component analysis (PCA) of AT2 cells from WT, HPS1, HPS2, and HPS1/2 mice at 8 weeks of age. (**C**) Differential expression analysis of AT2, transitional, and AT1 cell genes and p53 signaling pathway and cellular senescence genes in AT2 cells from WT, HPS1, HPS2, and HPS1/2 mice at 8 weeks of age. (**D**) Overall gene set score for cellular senescence. (**E**) Representative β-galactosidase staining of AT2 cells isolated from WT and HPS1/2 mice at 8 and 48 weeks of age. (**F**) Quantification of the percentage of β-galactosidase^+^ cells in WT, HPS1, HPS2, and HPS1/2 mice at 48 weeks of age. DAPI stains nuclei (blue). All quantification data are represented as mean ± SEM. Statistics using 2-tailed unpaired Student’s *t* tests: ** *P* < 0.01. *n* = 4–8 per group for bulk mRNA sequencing, *n* = 3 per group for β-galactosidase staining. Scale bars in **E**, 50 μm.

Given the aberrant differentiation and impaired proliferation exhibited by HPS AT2 cells, we sought to identify pathways that could underlie impaired AT2 cell progenitor function and connect them with mechanisms known to drive pulmonary fibrosis. As we observed these changes in progenitor cell function early in the disease course, prior to the onset of fibrosis, we examined the transcriptomes of AT2 cells isolated from WT, HPS1, HPS2, and HPS1/2 mice at 8 weeks of age (Figure 5A). Fluorescence-activated cell sorting (FACS) of AT2 cells revealed a significant decrease in the percentage of AT2 cells captured from HPS mice compared with WT (Supplemental Figure 7, A–D). Principal component analysis (PCA) revealed significantly different transcriptomes in AT2 cells from WT, HPS1, HPS2, and HPS1/2 mice (Figure 5B). Differential gene expression testing and overrepresentation analysis demonstrated upregulation of genes associated with immune cell activation and cell cycle regulation in HPS AT2 cells (Supplemental Figure 8, A–J). HPS AT2 cells displayed decreased expression of canonical markers of AT2 cells, increased expression of AT1 cell markers, as well an increase in expression of genes associated with a reprogrammed transitional cell state, including *Krt8*, *Cldn4*, *Lgals3*, and *Krt19* (Figure 5C) (16–18). These observations prompted us to examine markers of p53 signaling and cellular senescence, including *Trp53*, *Cdkn1a*, *Cdkn2d*, and *Ccnd1*, which were upregulated in HPS1, HPS2, and HPS1/2 AT2 cells, with the greatest change in expression seen in HPS1/2 AT2 cells (Figure 5, C and D) (19–21). In addition, by 48 weeks of age, when significant fibrotic disease is present, 5.9% ± 1.8% of AT2 cells isolated from HPS1/2 mice stained positive for β-galactosidase, a marker for senescent cells. In contrast, β-galactosidase activity was not detected in AT2 cells from WT or HPS mice at 8 weeks of age or WT, HPS1, or HPS2 mice at 48 weeks of age (*P* = 0.005) (Figure 5, E and F).

### HPS AT2 cells are primed to enter a p53-mediated Krt8^+^ reprogrammed transitional cell state and are present in lung tissue from a patient with HPS-1.

**Figure 6:**
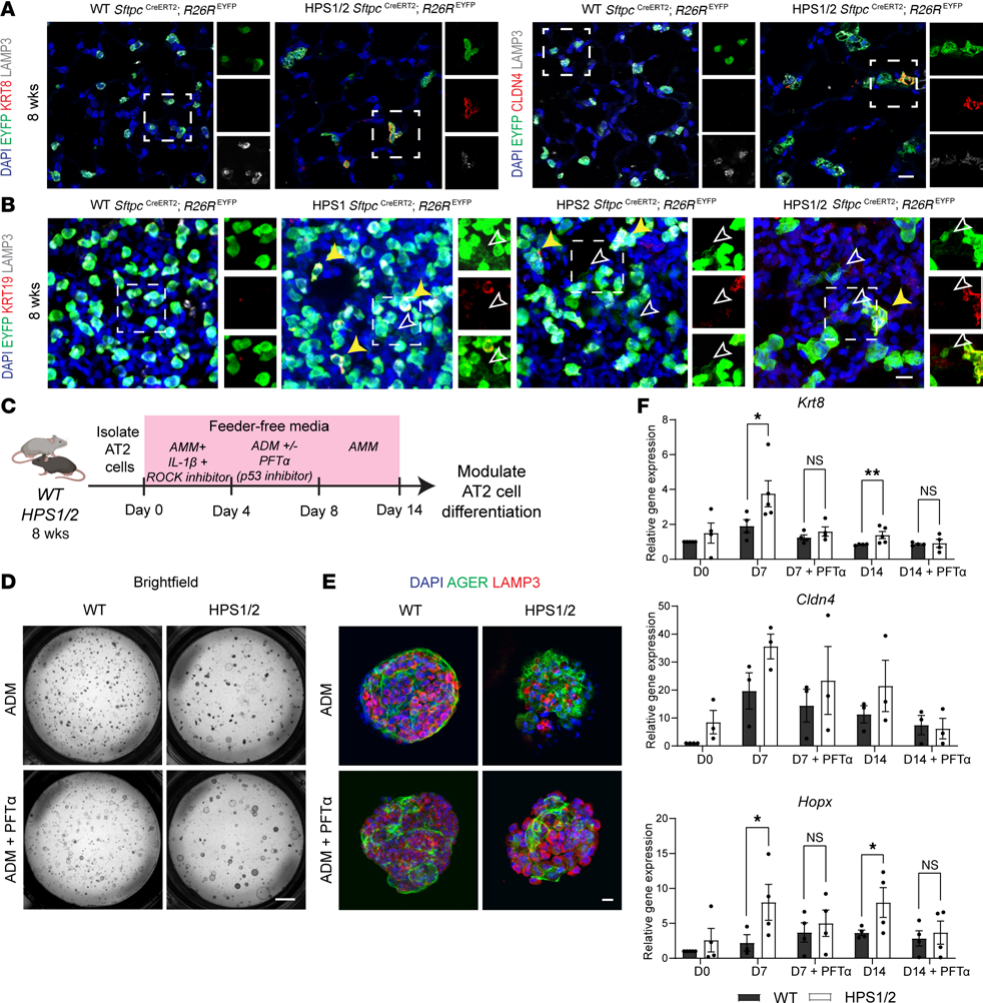
A p53-mediated Krt8^+^ reprogrammed transitional cell state in HPS mice. (**A**) IF staining of paraffin-embedded lung tissue for EYFP, KRT8 or CLDN4, and LAMP3 in WT and HPS1/2 *Sftpc*^CreERT2/+^ *R26R*^EYFP/+^ mice at 8 weeks of age. Dashed line boxes represent EYFP^+^ cells with inset boxes displaying EYFP, KRT8 or CLDN4, and LAMP3 staining. (**B**) IF staining of whole-mount lung tissue for EYFP, KRT19, and LAMP3 in WT, HPS1, HPS2, and HPS1/2 *Sftpc*^CreERT2/+^ *R26R*^EYFP/+^ mice at 8 weeks of age. White arrows indicate squamous EYFP^+^KRT19^–^LAMP3^–^ AT2-derived AT1 cells, and yellow solid arrows indicate cuboidal EYFP^+^KRT19^+^ cells; dashed line boxes and insets display EYFP and KRT19 and merged images. (**C**) Schematic for alveolosphere culture system with feeder-free alveolar maintenance media (AMM) and alveolar differentiation media (ADM) and treatment with pifithrin-α (PFTα). (**D**) Bright-field imaging and (**E**) IF staining of alveolospheres for AGER and LAMP3 generated with AT2 cells from WT or HPS1/2 mice with and without treatment with PFTα at day 14 (D14) of culture. (**F**) Relative gene expression of selected reprogrammed transitional cell (*Krt8*, *Cldn4*) and AT1 cell (*Hopx*) genes in alveolospheres at day 0 (D0), day 7 (D7), and day 14 (D14) of culture with and without treatment with PFTα. Relative gene expression compared with WT at D0. Each point represents 4 replicate wells per time point for a total of *n* = 3–4 mice. DAPI stains nuclei (blue). All quantification data are represented as mean ± SEM. Statistics using 2-tailed unpaired Student’s *t* tests: * *P* < 0.05; ** *P* < 0.01. Scale bars in **A**, **B**, and **E** 20 μm (inset boxes equals to main image); **D** 1 mm. Schematic created with BioRender.com.

With the aberrant differentiation exhibited by HPS AT2 cells and transcriptomic evidence of a reprogrammed transitional cell state, we sought to identify this cell state in situ. In HPS1/2 *Sftpc*^CreERT2/+^ *R26R*^EYFP/+^ mice, the previously captured lineage-labeled cuboidal EYFP^+^LAMP3^–^ cells (Figure 4D) demonstrated coexpression of markers of a reprogrammed transitional cell state, including KRT8, CLDN4, LGALS3, and KRT19, starting at 8 weeks and accumulating through 24 weeks of age (Figure 6, A and B, and Supplemental Figure 9, A–C). While KRT8^+^ cells were not detected in WT, HPS1, or HPS2 *Sftpc*^CreERT2/+^ *R26R*^EYFP/+^ mice, KRT19^+^ cells were present in HPS1 and HPS2 *Sftpc*^CreERT2/+^ *R26R*^EYFP/+^ mice at 8 weeks of age (Figure 6, A and B, and Supplemental Figure 9D). In all HPS models, squamous lineage-labeled EYFP^+^LAMP3^–^ cells did not express KRT19, suggesting they were AT2-derived AT1 cells rather than reprogrammed transitional cells (Figure 6B).

To further investigate the dynamics of AT2 cell differentiation and of this Krt8^+^ reprogrammed transitional cell state, we utilized an in vitro feeder-free alveolosphere culture system to evaluate cell-intrinsic differentiation (Figure 6C) (22, 23). Alveolospheres were generated with AT2 cells from WT, HPS1, HPS2, and HPS1/2 mice at 8 weeks of age, producing AT1 cells as indicated by AGER staining (Figure 6, D and E). Upon induction of differentiation, *Krt8*, *Cldn4*, and *Hopx* expression increased across all groups. Expression of *Krt8* and *Hopx* was significantly higher in HPS1/2 alveolospheres compared with WT on day 7 and continued to be significantly upregulated compared with WT on day 14 (Figure 6F). As transcriptomic data suggested this aberrant differentiation could be driven by p53 activation, alveolospheres were treated with PFTα, a p53 inhibitor, during induction of differentiation. Treatment with PFTα decreased *Krt8* and *Hopx* expression in HPS1/2 alveolospheres on day 7 and day 14 of culture to levels comparable to those in WT alveolospheres (Figure 6F). Similar trends in gene expression were seen in HPS1 and HPS2 alveolospheres compared with WT (Supplemental Figure 10, B–D). Expression of *Sftpc* was suppressed across all groups in alveolosphere culture; with induction of differentiation, increases in *Sftpc* expression after treatment with PFTα were not statistically significant (Supplemental Figure 10A).

**Figure 7:**
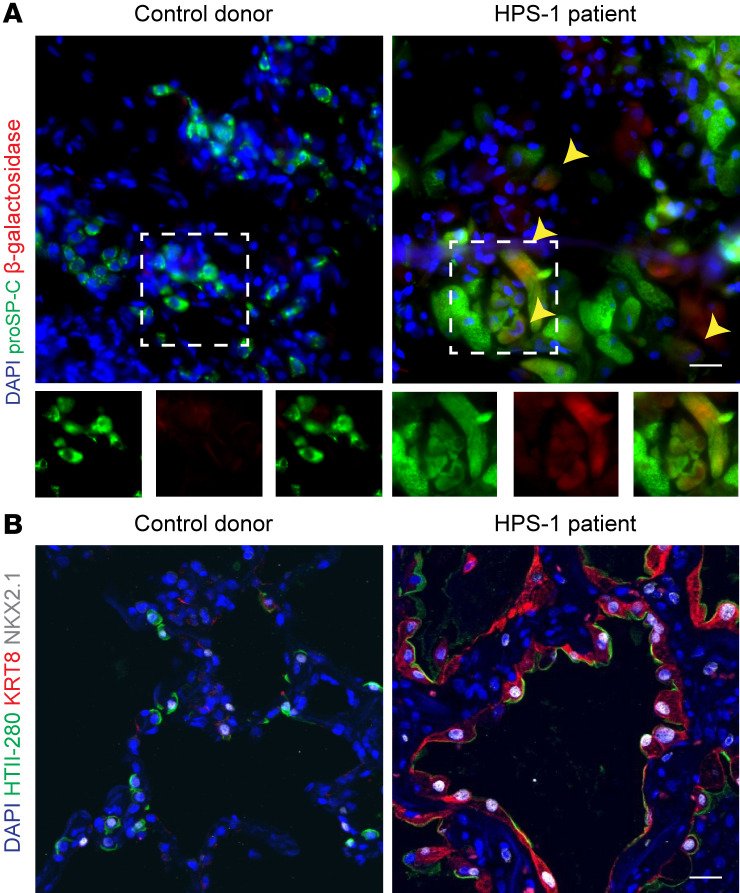
Senescence and a Krt8^+^ reprogrammed transitional cell state in a patient with HPS-1. (**A**) IF staining of paraffin-embedded lung tissue from an age- and sex-matched control donor patient and HPS-1 patient for proSP-C and β-galactosidase. Arrows and dashed line boxes represent proSP-C^+^ cells with inset boxes displaying proSP-C and β-galactosidase staining with merged images. (**B**) IF staining of paraffin-embedded lung tissue from an age- and sex-matched control donor patient and HPS-1 patient for HTII-280, KRT8, and NKX2.1. DAPI stains nuclei (blue). All scale bars, 20 μm (inset boxes equal scale to main image).

Finally, explanted lung tissue from a patient with HPS-1 pulmonary fibrosis revealed proSP-C^+^ AT2 cells that stained positive for β-galactosidase (Figure 7A). KRT8-expressing cells that coexpressed an epithelial cell marker, NKX2.1, were also detected (Figure 7B). A subset of these KRT8^+^NKX2.1^+^ cells also expressed HTII-280, a human AT2 cell marker (Figure 7B). Neither β-galactosidase^+^ AT2 cells nor KRT8^+^ epithelial cells were identified in lung tissue from age-matched and sex-matched control donor patients (Figure 7, A and B). Taken together, these results demonstrate that HPS AT2 cells are primed to differentiate and persist in a Krt8^+^ reprogrammed transitional cell state, which can be ameliorated in vitro in HPS1/2 mice by inhibiting the activity of p53.

## Discussion

**Figure 8:**
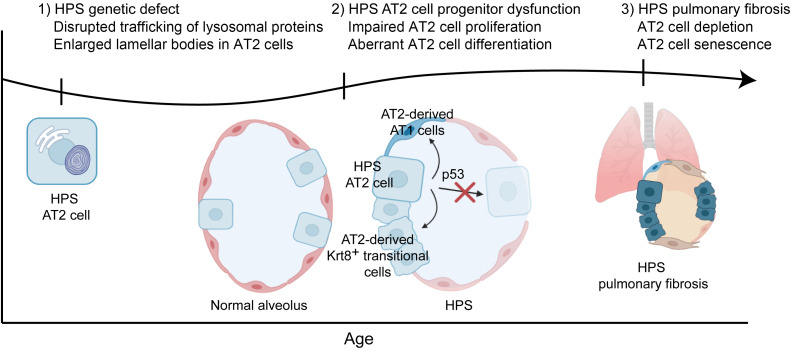
Time course of AT2 cell dysfunction in HPS-PF. Our current working hypothesis is that the HPS genetic mutations disrupt AT2 progenitor cell function early, with activation of the p53 pathway, impairing AT2 cell proliferation and driving aberrant AT2 cell differentiation. Over time, this results in accelerated AT2 cell loss with senescence and depletion of the stem cell pool and progressive fibrotic remodeling.

As a monogenic disorder with murine models that recapitulate key features of the disease, HPS presents a unique opportunity for the study of progressive pulmonary fibrosis. Although the lysosomal and endosomal protein trafficking defects in HPS have been well characterized, the link between these dysregulated cellular processes and the downstream functional consequences for the alveolar epithelium and their role in fibrotic remodeling remains incompletely understood. In addition, there has been debate about whether mechanisms in HPS-PF are distinct from other heritable forms of pulmonary fibrosis and idiopathic pulmonary fibrosis (24, 25). In this study, we demonstrate early disruption of alveolar epithelial maintenance in HPS in the setting of AT2 cell progenitor dysfunction. We propose that over time, impaired AT2 cell proliferation and aberrant differentiation contribute to progressive AT2 cell loss, and ultimately, senescence and depletion of the stem cell pool with pulmonary fibrosis (Figure 8). Intrinsic AT2 progenitor cell dysfunction is a potentially novel mechanism in HPS-PF primed for early intervention and highlights convergent mechanisms of disease between HPS-PF and other forms of pulmonary fibrosis.

Under homeostatic conditions, AT2 cells are largely quiescent, yet some degree of proliferation is required to maintain the alveolar epithelium (26). Other models have demonstrated that interruption of AT2 cell proliferative capacity contributes to fibrotic remodeling (19, 27). Although we observed similarly low rates of AT2 proliferation in WT and HPS mice under basal conditions, the decreased rates of proliferation in HPS AT2 cells were striking in multiple experimental conditions, including in an ex vivo lung organoid model and in 2 in vivo models, after mitogenic stimulation and with an influenza injury model. This impaired proliferation may result from not only depletion of the AT2 cell progenitor pool but also decreased proliferative capacity of these progenitor cells as a result of the HPS mutations. Notably, HPS1 AT2 cells appeared to perform comparably to WT AT2 cells in an organoid model but exhibited impaired proliferation after administration of KGF and after injury in an influenza model. This may reflect differences in the consequences of the HPS1 and HPS2 mutations on AT2 cell function as well as the models utilized to study cell proliferation and regeneration (10, 28). Furthermore, differences in AT2 cell regenerative capacity by injury severity after acute influenza infection suggest that the HPS mutations have divergent effects on AT2 cell subpopulations, particularly those that function as progenitor cells (15, 29).

In addition to impaired proliferation, HPS AT2 cell progenitor dysfunction appears to also manifest with aberrant differentiation. In adults, AT2-to-AT1 cell differentiation is typically observed in the setting of injury, as a subset of progenitor cells are mobilized to regenerate the alveolar epithelium (15, 30). This process occurs at very low rates during homeostasis, with an approximate turnover rate of 0.005% of the alveolar surface with new AT2-derived AT1 cells per day (31). In bleomycin models of pulmonary fibrosis, epithelial cell subpopulations enriched in expression of *Krt8*, *Cldn4*, *Lgals3*, and *Krt19*, which have been referred to as pre-alveolar type 1 transitional cell state, damage-association transition progenitors, and Krt8^+^ alveolar differentiation intermediates, appear to emerge from incomplete differentiation of AT2 cells (16–18). In HPS, through lineage tracing and alveolosphere studies without exogenous stimuli, we demonstrate that AT2 cells from HPS1, HPS2, and HPS1/2 mice display aberrant differentiation. The most severe phenotype with the emergence of a Krt8^+^ reprogrammed transitional cell state was observed in HPS1/2 mice, a model system associated with spontaneous fibrosis, underscoring the potential role of these cells in fibrotic remodeling. Our data further support emerging evidence that this state may not necessarily be “transitional” in the sense that cells may lose their capacity to differentiate into AT1 cells. For some cells, entry into this state may be unidirectional, resulting in loss of the ability to contribute to functional alveolar epithelial maintenance and repair (32).

Given the widespread and nonselective injury induced by bleomycin, the contribution of other cell types and potential AT1 cell dysfunction in driving aberrant AT2 cell differentiation and the emergence of the Krt8^+^ reprogrammed transitional cell state has been debated (33). In subsequent studies, this cell state has been identified in genetic models with selective AT2 cell modification, including models of accelerated senescence of AT2 cells via *Trp53* activation, surfactant metabolism dysfunction (*Sftpc^C185G^* mice), and Nkx2.1 ablation in alveolar epithelial progenitors (19, 34, 35). Here, in a feeder-free alveolosphere culture system with only AT2 cells and no stromal support cells, HPS AT2 cells appear primed to differentiate and persist in this Krt8^+^ reprogrammed transitional cell state. Taken together, this suggests that aberrant differentiation in HPS is driven by intrinsic AT2 cell dysfunction, although other cell types may provide additional feedback to further amplify this process (32).

In the setting of impaired progenitor function and progressive AT2 cell loss, transcriptomic analyses highlighted a role for p53 activation in driving AT2 cell dysfunction and pulmonary fibrosis in HPS. From the study of telomere disorders, p53-mediated senescence of AT2 cells has long been implicated in pulmonary fibrosis (36). In an experimental murine model, conditional loss of function of SIN3 transcription regulator homolog A (*Sin3a*), a regulator of Trp53 acetylation, in AT2 cells induced senescence, impaired proliferation, and caused loss of AT2 cells, resulting in spontaneous, progressive fibrosis. Mice with loss of function of both *Sin3a* and p53 did not exhibit AT2 cell loss and did not develop significant fibrosis, indicating that activation of the p53 pathway is necessary to induce AT2 cell senescence and fibrosis (19). Previous studies of the Krt8^+^ transitional reprogrammed cell state have demonstrated that these cells show increased expression of genes involved in p53, DNA damage, and cellular senescence pathways (17, 19, 32). Indeed, inhibition of p53 was sufficient to attenuate upregulation of *Krt8* and *Hopx* in alveolospheres generated with HPS AT2 cells compared with WT, suggesting that p53 activation is the driver of aberrant differentiation and this transitional cell state in HPS. Prior studies have demonstrated that HPS AT2 cells have excess production of monocyte chemoattractant protein–1 (MCP-1), a senescence-associated secretory phenotype factor, and epithelial deletion of MCP-1 reduces fibrotic susceptibility in HPS mice (12, 37). Patients with HPS-1 have also been shown to have elevated levels of MCP-1 in bronchoalveolar lavage fluid that correlate with lung disease severity (38). Additional studies are required to evaluate the secretome of HPS AT2 cells, as well as further examine interactions between AT2 cells, macrophages, and fibroblasts, to identify which factors drive fibrotic remodeling in HPS.

Although increased apoptosis of AT2 cells has been observed across many forms of pulmonary fibrosis, there remains debate about whether depletion of AT2 cells is necessary or sufficient to drive fibrotic remodeling (25, 39–41). Using diphtheria toxin–mediated epithelial cell ablation in SPC-diphtheria toxin receptor mice, Sisson et al. showed that AT2 cell loss resulted in fibrotic remodeling. When Yao et al. selectively ablated AT2 cells in *Sftpc*^CreER^ *Rosa*^DTA^ *Rosa*^mTmG^ mice, significant AT2 cell loss and collagen deposition were observed, but fibrosis diminished over time, suggesting a transient effect. In contrast, progressive pulmonary fibrosis was observed in *Sin3a* loss-of-function mice with AT2 cell senescence in addition to AT2 cell loss (19). Although previous studies have described increased AT2 cell apoptosis in HPS1/2 mice as well as in bleomycin-challenged but not naive HPS1 and HPS2 single-mutant mice, we did not detect AT2 cell apoptosis in any of the HPS models up to 48 weeks of age (8, 10). In addition to differences in techniques and assays used to evaluate apoptosis, our ability to capture rare events may have been limited without a stereotactic approach, and we cannot rule out that other mechanisms of increased cell death, including necroptosis, may also contribute to AT2 cell loss in HPS.

The exact mechanisms by which the HPS endosomal trafficking mutations result in p53 activation and AT2 progenitor cell dysfunction are unknown. There have been several prior reports demonstrating the emergence of a reprogrammed transitional cell state with lung injury and with disrupted proteostasis (42–46). While the HPS mutations could produce cell cycle defects that directly result in cell cycle arrest, it is also possible that they contribute to other forms of cellular dysfunction, including mitochondrial dysfunction and aberrant autophagy, which can induce cellular senescence (6, 9). As these mechanisms have been implicated in other fibrotic lung diseases, there may be interconnecting pathways contributing to AT2 cell dysfunction in HPS. In mice, there may be an additive effect of the HPS1 and HPS2 mutations that results in markedly impaired progenitor cell function and accelerated senescence with the emergence of a Krt8^+^ reprogrammed transitional cell state in double-mutant HPS1/2 AT2 cells. This could underlie the divergent phenotypes observed, with fibrotic sensitivity in HPS1 and HPS2 mice in comparison with the development of spontaneous, progressive fibrosis in HPS1/2 mice. It is important to note, however, that patients only bear single variants, either in *HPS1* or in *HPS2*. The emergence of KRT8^+^ epithelial cells and spontaneous pulmonary fibrosis in human HPS patients is likely to result from the combined effects of genetic and environmental factors on AT2 cell function.

Our findings reveal intrinsic AT2 progenitor cell dysfunction in HPS, occurring early in the disease course prior to the onset of fibrosis and suggesting the potential for early targeted intervention. In addition, our findings reveal common pathways driving the fibrotic response across sporadic and genetic forms of pulmonary fibrosis. The relative contributions of rare and common genetic risk variants, in conjunction with environmental exposures, may vary across different forms of pulmonary fibrosis, but it is likely that these pathways converge, resulting in failure to maintain alveolar epithelial integrity. Understanding the mechanisms that drive AT2 cell differentiation in HPS-PF and the optimal method to modulate the p53 pathway to promote epithelial maintenance and repair and prevent fibrotic remodeling will be critical. Additional studies at single-cell resolution may enable further characterization of the subpopulations of epithelial cells in HPS. We also found features of functional divergence between HPS1 and HPS2 AT2 cells in an organoid model and an influenza injury model, highlighting that the differences that exist between the HPS subtypes may help illuminate key aspects of HPS biology and have clinical relevance. Given the shared pathways that have been identified, further study of the role of alveolar epithelial dysfunction in HPS-PF should have broad applicability across genetic and sporadic fibrotic lung diseases.

## Methods

### Sex as a biological variable.

For studies involving mice, both male and female mice were utilized, as fibrosis occurs equally in patients with HPS of both sexes.

### Animal studies and treatment.

Naturally occurring HPS1, HPS2, and HPS3 mice, which harbor mutations in the mouse homologs of the *Hps1*, *Ap3b1* (*Hps2*), and *Hps3* genes, respectively, were maintained on the C57BL6/J background (47). HPS2 TG mice, with partial correction of AP3b1 in the lung epithelium driven by human SFTPC promoter, were as described previously (2). Double-mutant HPS1/2 mice were generated by mating homozygous HPS1 and homozygous HPS2 mice (11, 13, 48). WT *Sftpc*^CreERT2/+^ *R26R*^EYFP/+^ (Jackson Laboratory, strain 006148) mice were provided by in-house and generated by Harold Chapman (University of California, San Francisco, San Francisco, California, USA), as described previously, and bred with HPS1, HPS2, and HPS1/2 mice to generate HPS1 *Sftpc*^CreERT2/+^ *R26R*^EYFP/+^, HPS2 *Sftpc*^CreERT2/+^ *R26R*^EYFP/+^, and HPS1/2 *Sftpc*^CreERT2/+^ *R26R*^EYFP/+^ mice, respectively (49). We used 4- to 48-week-old animals for experiments, as specified. For lineage trace experiments, 4-week-old *Sftpc*^CreERT2/+^ *R26R*^EYFP/+^ mice were administered tamoxifen (200 mg/kg body weight) in corn oil via oral gavage for 3 consecutive days. KGF was obtained as palifermin (Amgen). KGF dissolved in vehicle (PBS) at a dose of 5 mg/kg or vehicle alone was administrated via intratracheal injection 48 hours prior to euthanasia and isolation of lungs (50). For cell proliferation experiments, pulse-chase EdU dissolved in filtered PBS was administered via intraperitoneal injection at a dose of 50 mg/kg 4 hours prior to euthanasia and isolation of lungs.

### Human lung tissue.

Healthy lung samples were obtained through the University of Pennsylvania Lung Biology Institute’s Human Lung Tissue Bank. These samples were obtained from deidentified unused lungs donated for organ transplantation through an established protocol (Prospective Registry of Outcomes in Patients Electing Lung Transplantation [PROPEL], approved by University of Pennsylvania Institutional Review Board [IRB]) with written informed consent in accordance with institutional and NIH procedures, and consent was provided by next of kin or health care proxy (51). HPS-1 patient tissue samples were obtained through the University of Pennsylvania as part of the PROPEL and Vanderbilt University (51). The University of Pennsylvania and Vanderbilt University IRBs approved this study, and written informed consent was obtained. All patient information was removed before use.

### Histology and IF.

Lungs were harvested at a constant pressure of 30 cm H_2_O and were fixed overnight at 4°C in 4% paraformaldehyde (PFA). For whole-mount imaging, lung tissue was washed in PBS, embedded in 4% low-melting agarose, and sectioned at 150 μm thickness. For paraffin embedding, lung tissue was washed in PBS, dehydrated in a graded series of ethanol washes, embedded in paraffin wax, and sectioned at 7 μm thickness. For human tissue, tissue was cut from the distal regions of the human lungs. We washed 2 by 2 cm pieces of peripheral parenchyma 5 times in cold PBS and then placed them in 2% PFA overnight. The tissue was washed with PBS and dehydrated to 100% ethanol over the subsequent 12 to 24 hours, paraffin-embedded, and then sectioned at a thickness of 8 μm. H&E and Masson’s trichrome staining were performed according to standard procedures. IF staining was performed with antibodies for proSP-C (Invitrogen, PA5-71680), NKX2.1 (Invitrogen, MA5-13961), GFP (Abcam, ab6673), LAMP3 (Synaptic Systems, 391 005), AGER (R&D Systems, Bio-Techne, MAB1179), KRT8 (DSHB), CLDN4 (Invitrogen, PA5-32354), LGALS3 (Invitrogen, 14-5301-82), KRT19 (Invitrogen, SA30-06), and HTII-280 (Terrace Biotech). EdU staining was performed with the Click-iT EdU cell proliferation kit according to the manufacturer’s instructions (Invitrogen). TUNEL staining was performed using TUNEL assay apoptosis detection kit per the manufacturer’s instructions (Biotium). Slides were mounted in VECTASHIELD Antifade Mounting Medium (Vector Labs). Images were captured using a Leica confocal microscope. All image quantification was performed on serial confocal images from *Z*-stack maximum-intensity composites. For quantification, 10 images per sample were randomly selected via the 405 nm (DAPI) channel. Manual counting of DAPI^+^ nuclei, proSP-C^+^ cells, NKX2.1^+^ cells, and EYFP^+^ cells was performed in a blinded fashion.

### AT2 cell isolation.

Murine lung single-cell suspensions were prepared by dispase digestion (52). Briefly, dispase (50 U/mL) was instilled in lungs followed by agarose to ensure digestion of distal airspaces and prevent incorporation of epithelial cells from distal airways. Lungs were incubated in a dispase solution for 45 minutes at room temperature, DNase I (STEMCELL Technologies) was added, and lungs were teased apart with forceps. Serial filtration through 70 μm and 40 μm cell strainers (Falcon, Corning) was performed to create a single-cell suspension, followed by ACK lysis to remove red blood cells. AT2 cells were subsequently isolated by FACS using antibodies for cell surface markers via negative selection for CD45, positive selection for EpCAM, and negative selection for β4 integrin (CD104) (BioLegend) (14, 49). Samples of the AT2 cell suspensions were fixed and stained for proSP-C, with confirmation of purity greater than 95%.

### Lung organoid assays.

AT2 cells were isolated as described above. Live cell numbers were verified using a hemocytometer and trypan blue solution. In each technical replicate, 5,000 AT2 cells were combined with 50,000 primary outgrowth WT murine lung fibroblasts in 50% Matrigel (growth factor reduced, phenol red free) (Corning) and in small airway growth media (Lonza) with the following additives: insulin, transferrin, bovine pituitary extract, retinoic acid, and gentamicin (all from Lonza), with cholera toxin (MilliporeSigma) and FBS (Corning). The ROCK inhibitor thiazovivin (STEMCELL Technologies) was included in the medium for the first 2 days, and medium was changed every 2 to 3 days. Images were captured over the course of culture using a Leica Thunder microscope. Images were processed in ImageJ (NIH) with thresholding and then binarized. All organoids within a well were used for quantification. Organoid number and size were quantified using the analyze particles function in ImageJ with a cutoff of 1,500 μm^2^. CFE was calculated by dividing the organoid number by the number of input AT2 cells, 5,000. Organoids were fixed and stained with antibodies for proSP-C (Invitrogen, PA5-71680) and AGER (Rage) (R&D Systems, Bio-Techne, MAB1179).

### Influenza infection.

H1N1 PR8 influenza virus was provided by Andrew Vaughan (University of Pennsylvania), and titration by 50% tissue culture infectious dose for infectivity was performed in MDCK cells (ATCC) (53). For AT2 cell proliferation studies, we performed an initial set of experiments with WT and HPS2 mice, aged 8 to 12 weeks. Mice were anesthetized with 4% isoflurane in 100% O_2_ via an anesthesia vaporizer system, and H1N1 PR8 influenza was delivered intratracheally at a dose of 100 U diluted in 50 μL PBS. In a subsequent experiment, HPS1 and WT mice, aged 8 to 12 weeks, were anesthetized with xylazine and ketamine via intraperitoneal injection, and H1N1 PR8 influenza was delivered intranasally at a dose of 100 U diluted in 50 μL PBS. For weight and oxygen saturation measurements, a subsequent cohort of WT, HPS1, and HPS2 mice, aged 8 to 12 weeks, were also anesthetized with xylazine and ketamine via intraperitoneal injection, and H1N1 PR8 influenza was delivered intranasally at a dose of 100 U diluted in 50 μL PBS. Mouse weights were tracked daily starting from the day of infection. Mice were shaved, and pulse oximetry measurements were obtained using MouseOx Plus (Starr Life Sciences) every second or third day starting from the day of infection for the time points noted.

### Lung injury assessment.

To define regions of lung injury after influenza infection, an unbiased image classification machine learning model was used on H&E-stained sections to cluster image pixels by staining intensity, similar to previously described methodology (15, 29). Three regions of injury were defined: severely injured, mildly injured, and unaffected.

### Bulk mRNA sequencing.

Total RNA was extracted from freshly isolated AT2 cells using the RNeasy Micro Kit (QIAGEN) and stored at –80°C. Frozen total RNA was sent to GENEWIZ/Azenta Life Sciences for bulk mRNA sequencing, using their standard RNA-Seq service. Reads in FASTQ format were aligned to the National Center for Biotechnology Information (NCBI) mouse genome (mm10/GRCm38) and quantified at the gene level using the nf-core/rnaseq v3.8.1 Nextflow pipeline (54, 55). Differential gene expression analysis was performed using DESeq2 and scanpy (56, 57). Aggregate gene set scores, including the REACTOME cellular senescence score, were calculated on a per-sample basis by selecting genes in the corresponding gene set and scoring them using the JASMINE scoring metric (58).

### β-galactosidase staining.

Murine AT2 cells were isolated as described above. AT2 cells were deposited on glass slides by cytospin centrifugation at 28*g* for 5 minutes. Fixation and staining were performed using a β-galactosidase staining kit per the manufacturer’s instructions at pH 6.0 (Cell Signaling Technology). Cells were counterstained with DAPI to enable quantification. Human HPS-1 lung tissue was fresh frozen and sectioned via cryotome. Fixation and staining were performed using a β-galactosidase staining kit per the manufacturer’s instructions (Invitrogen, C10851). Sections were then counterstained for proSP-C (Abcam) and DAPI. The images were captured using a Keyence inverted fluorescence microscope.

### Alveolosphere differentiation assay.

Alveolosphere culture was performed as previously described (22, 23). AT2 cells were isolated as described above. Live cell numbers were verified using a hemocytometer and trypan blue solution. In each technical replicate, 7,000 AT2 cells were plated in 50% Matrigel (growth factor reduced, phenol red free) (Corning). Alveolospheres were cultured in alveolar maintenance medium with IL-1β and the ROCK inhibitor Y-27632 (MilliporeSigma) for the first 4 days, alveolar differentiation medium for the next 4 days, and then transitioned to alveolar maintenance medium for the remaining 6 days in culture. Media were changed every 2 to 3 days. PFTα was added to the alveolar differentiation medium at a concentration of 30 μM (59, 60). Organoids were fixed and stained with antibodies for LAMP3 (Synaptic Systems, 391 005) and AGER (Rage) (R&D Systems, Bio-Techne, MAB1179).

### Quantitative polymerase chain reaction.

Alveolospheres were dissociated from Matrigel using Cell Recovery Solution (Corning). RNA was isolated from alveolospheres using Direct-zol RNA Microprep Kits (Zymo). cDNA was synthesized from total RNA using High-Capacity cDNA Reverse Transcription Kits (Applied Biosystems). Quantitative polymerase chain reaction (qPCR) was performed using the TaqMan system (Applied Biosystems). qPCR primers are listed in Supplemental Table 1. Relative gene expression was calculated using the ΔΔCt method (61).

### Picrosirius red staining.

Staining for collagen was performed using picrosirius red staining kit per the manufacturer’s instructions (Polysciences Inc.). Digital morphometric measurements and collagen quantitation were performed as previously described (62). WT lung tissue was normalized to a collagen area of 3.0%.

### Statistics.

Statistical analyses were performed with GraphPad Prism. Two-tailed unpaired Student’s *t* tests were used for comparison between groups for parametric data and Mann-Whitney *U* test for nonparametric data. Results are presented as mean ± SEM. *P* < 0.05 was considered significant. For multiple comparisons, the Benjamini-Hochberg procedure was utilized with a false discovery rate of 0.05. Adjusted *P* values < 0.05 were considered significant.

### Study approval.

All procedures for animal experiments were performed under the guidance of and in compliance with all ethical regulations of the Children’s Hospital of Philadelphia Institutional Animal Care and Use Committee. Human HPS-1 specimens were obtained at the time of lung transplantation under established protocols at the University of Pennsylvania 458 (IRB protocol 813685) and Vanderbilt University (IRB protocol 171657). The normal human donor samples used were from deidentified, unused lungs donated for research (PROPEL, IRB protocol 813685) with approval of the University of Pennsylvania and Vanderbilt University IRBs and in accordance with NIH procedures (51). Written informed consent was provided by next of kin or health care proxy.

### Data availability.

Mouse AT2 cell bulk RNA-sequencing data are available in the NCBI’s public functional genomics data repository Gene Expression Omnibus (accession number GSE235628). Values for all data points in graphs are reported in the Supporting Data Values file.

## Author contributions

JYW designed and performed experiments, analyzed and interpreted the data, and wrote and edited the manuscript. SNM analyzed and interpreted the data and edited the manuscript. SS designed and performed experiments and edited the manuscript. BJB designed experiments and edited the manuscript. RJ and JJG performed experiments and reviewed the manuscript. SML, MCB, and EC provided access to human samples and assisted with tissue processing. JBK designed experiments, provided access to human samples, and assisted with tissue processing. JAK, JAZ, and DBF designed experiments, interpreted the data, and edited the manuscript. LRY designed the study, interpreted the data, and wrote and edited the manuscript.

## Supplementary Material

Supplemental data

Supporting data values

## Figures and Tables

**Table 1:**
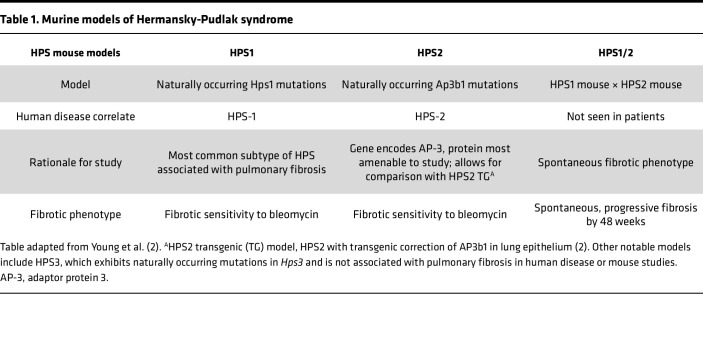
Murine models of Hermansky-Pudlak syndrome

## Version 1 12/19/2024

In-Press Preview

## Version 2 02/10/2025

Electronic publication
